# Droplet Digital PCR Based Androgen Receptor Variant 7 (AR-V7) Detection from Prostate Cancer Patient Blood Biopsies

**DOI:** 10.3390/ijms17081264

**Published:** 2016-08-04

**Authors:** Yafeng Ma, Alison Luk, Francis P. Young, David Lynch, Wei Chua, Bavanthi Balakrishnar, Paul de Souza, Therese M. Becker

**Affiliations:** 1 Centre for Circulating Tumor Cell Diagnostics and Research, Ingham Institute for Applied Medical Research, 1 Campbell St., Liverpool, NSW 2170, Australia; yafeng.ma@unsw.edu.au (Y.M.); alison.luk@unsw.edu.au (A.L.); francis.young@student.unsw.edu.au (F.P.Y.); 18292682@student.westernsydney.edu.au (D.L.); P.DeSouza@westernsydney.edu.au (P.d.S.); 2 South Western Clinical School, University of New South Wales, Goulburn St., Liverpool, NSW 2170, Australia; 3 Western Sydney University Clinical School, Elizabeth St., Liverpool, NSW 2170, Australia; 4 Department of Medical Oncology, Liverpool Hospital, Elizabeth St & Goulburn St., Liverpool, NSW 2170, Australia; Wei.Chua2@sswahs.nsw.gov.au (W.C.); Bavanthi.Balakrishna@sswahs.nsw.gov.au (B.B.)

**Keywords:** biomarker, androgen receptor, AR-V7, prostate cancer, ddPCR, CTC

## Abstract

Androgen receptor splice variant V7 (AR-V7) was recently identified as a valuable predictive biomarker in metastatic castrate-resistant prostate cancer. Here, we report a new, sensitive and accurate screen for AR-V7 mRNA expression directly from circulating tumor cells (CTCs): We combined EpCAM-based immunomagnetic CTC isolation using the IsoFlux microfluidic platform with droplet digital polymerase chain reaction (ddPCR) to analyze total AR and AR-V7 expression from prostate cancer patients CTCs. We demonstrate that AR-V7 is reliably detectable in enriched CTC samples with as little as five CTCs, even considering tumor heterogeneity, and confirm detection of AR-V7 in CTC samples from advanced prostate cancer (PCa) patients with AR-V7 detection limited to castrate resistant disease status in our sample set. Sensitive molecular analyses of circulating tumor cells (CTCs) or circulating tumor nucleic acids present exciting strategies to detect biomarkers, such as AR-V7 from non-invasive blood samples, so-called blood biopsies.

## 1. Introduction

The insight that cancers, even of the same type, show strong inter- and intra-patient heterogeneity has emerged in recent years. Tumor biomarkers are most commonly conceptualized as specific cellular, biochemical or molecular alterations that characterize heterogeneous subcategories of cancers. Consequently, patient management increasingly relies on the detection of such tumor biomarkers to predict prognosis, guide therapies and monitor treatment response as well as the development of specific resistance mechanisms [1]. The number of identified actionable biomarkers (biomarkers that determine the best type of therapy) and the generation of novel targeted drugs are currently increasing faster than ever, leading to major changes in personalized therapy and clinical praxis.

Some serum proteins, such as the prostate specific antigen (PSA) in prostate cancer (PCa) and carcinoembryonic antigen (CEA) in colorectal cancer, are well established biomarkers and clinically widely used, although their correlation with other disease progression parameters is not always ideal [2,3]. More recently, there has been a trend towards analyzing cancer associated gene expression, mutations, amplifications or gene expression variants, which can be very specific, and may predict response and resistance to certain therapies. Some of these assays have already been validated and adopted as part of clinical practice. For instance the detection of v-Raf murine sarcoma viral oncogene homolog B (BRAF) V600E mutation is now decisive for melanoma treatment options that involve targeted BRAF kinase inhibitors, and V-Ki-ras2 Kirsten rat sarcoma viral oncogene homolog (KRAS) mutations in colorectal cancer patients predict resistance to anti-epithelial growth factor receptor (EGFR) monoclonal antibody based therapy (panitumumab and cetuximab) [4,5].

Traditionally, such molecular biomarkers are examined in excised tumor tissue or fine needle biopsies at a single timepoint in the disease course. However, such biopsies are not always available or informative [6]. To progress targeted therapies more broadly into the clinic, it is desirable to detect biomarkers from non-invasive, easily accessible biopsies that are ideally of modest cost. Additionally, repeated biopsies should be feasible to enable continuous monitoring of changes in biomarkers during treatment and development of drug resistance. To that end, blood biopsies are becoming increasingly attractive because various tumor-derived biomarkers can be screened from blood. Tumors release entire circulating tumor cells (CTCs) and circulating tumor nucleic acids (ctDNA and ctRNA) into the blood stream, and screening these for tumor associated genetic changes is becoming increasingly feasible [6,7]. While these kinds of assays still need further validation before they can be adopted into clinical practice, blood-based assays are extremely appealing to clinicians and researchers. Importantly these assays are known to detect evidence of the common tumor heterogeneity, which needs to be accounted for in biomarker analysis [8].

In PCa, genetic changes in the androgen receptor (AR), such as point mutation and gene amplification, render the receptor independent of upstream testosterone levels and cause resistance against androgen deprivation therapy (ADT), which is the predominant first line therapy for advanced disease [9]. Thus far, AR amplification screening using fluorescent in situ hybridization (FISH) and point mutation screening with PCR-based methods from CTC enriched samples have been reported [10,11,12,13]. AR transcriptional variants, for example, AR-V7 and AR-V567es, which encode constitutively active, truncated receptor proteins, cause ligand independent AR activation and are clinically relevant [14]. ADT-drug exposure rapidly induces AR-V7 expression in in vivo models and patient PCa cells, likely to compensate loss of regular AR signaling [9,15]. More importantly, the detection of AR-V7 in PCa CTCs has been correlated with metastatic castrate resistant prostate cancer (CRPC) and resistance against enzalutamide and abiraterone, and potentially superior clinical outcomes for patients on taxane therapy, though response to cabazitaxel has been shown to be independent of AR-V7 status [16,17,18]. Taken together, this suggests that AR-V7 may be a useful biomarker on which to base therapy initiation or therapy changes.

Here, we present development of a reliable droplet digital PCR based method to detect AR-V7 and total AR expression in PCa patient CTCs enriched by the IsoFlux system. Our assay has high specificity and sensitivity and detects AR-V7 expression in as little as five AR-V7 PCa cells spiked to produce a modeled CTC sample, and was confirmed in PCa patient CTCs.

## 2. Results and Discussion

### 2.1. Assay Optimization

**Figure 1:**
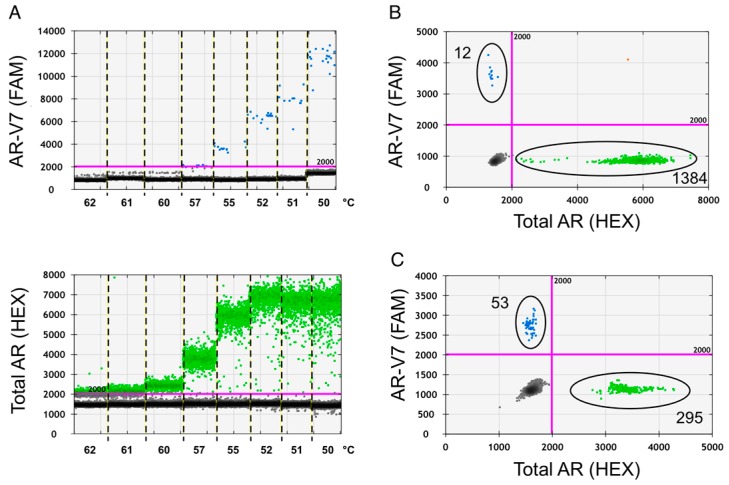
Annealing temperature optimization. Temperature gradient droplet digital PCR (ddPCR) was used to decide on the optimal annealing temperature for the assay. cDNA derived from 200 pg of cell line RNA was used per ddPCR reaction, multiplexed in the presence of probes and primers for both products. AR-V7 ddPCR products are shown in blue and total AR products in green. (**A**) Fluorescence product separation from background fluorescence from VCaP cell line AR-V7 FAM and total AR HEX reactions with indicated annealing temperatures is compared in 1-D graph presentation; (**B**) 2D separation of PCR products from VCaP; and (**C**) 22Rv1 prostate cancer (PCa) cell lines are depicted for optimized annealing temperature of 55 °C.

Droplet digital PCR (ddPCR), utilizing Taqman PCR principles, is a novel and sensitive method to detect rare mutations, gene expression and copy number variations in samples with limited amounts of nucleic acid templates [19]. To optimize the detection of total AR and AR-V7 transcript expression with ddPCR from total RNA we initially determined the appropriate annealing temperature for our assay using prostate cancer cell line total RNA as basis for ddPCR cDNA template synthesis. At 55 °C, annealing temperature for ddPCR reactions total AR and AR-V7 amplicon containing events (droplets) showed the best separation from empty baseline events and good PCR amplification was achieved (Figure 1).

### 2.2. Assay Specificity

To test assay specificity we determined total AR and AR-V7 expression in a cohort of PCa cell lines: 22Rv1, VCaP, C4-2, LNCaP, C4-2B, LAPC4, and PC3 and the b-lymphocyte line WME-099 (Table 1). Amongst the PCa cell lines, 22Rv1 showed high AR-V7 levels and AR-V7 versus total AR expression ratios (26%). This agrees with previous studies, using immunoblots to discriminate between full length AR and AR-V7 [20,21]. VCaP, known to carry AR gene amplification [22], expectedly expressed the highest level of total AR transcript, which was two to seven fold the level of other AR positive cell lines. LNCaP and its derivates, C4-2 and C4-2B, had consistently less than one AR-V7 copy per cell, and interestingly C4-2 expressed reduced levels of total AR transcript compared to the related C4-2B and parental LNCaP cells. LAPC4 had low levels of total AR expression, as previously reported [23], and no detectable AR-V7. PC3 cells are known to be AR-negative [24] and expectedly expressed neither total AR nor AR-V7 as did the control WME-099 lymphocytes. Similarly, we assayed healthy donor peripheral blood mononuclear cells (PBMCs) (5 male, 1 female sample), which had negligible total AR and no AR-V7 expression, confirming data from normal blood cells in previous reports (Table 2) [13,25].

### 2.3. Assay Sensitivity

**Figure 2:**
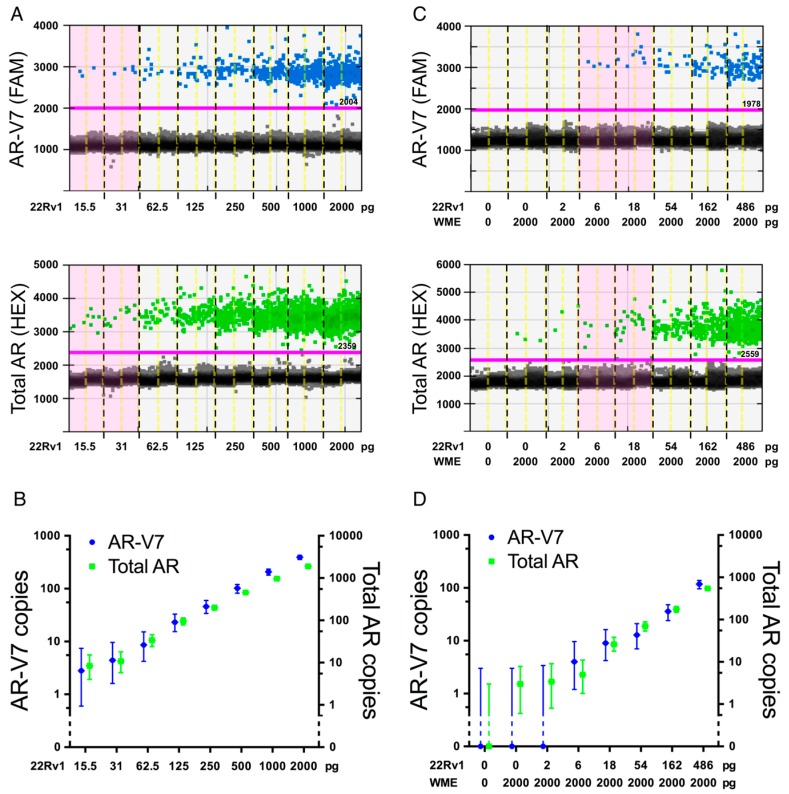
Sensitivity of AR-V7 detection. (**A**,**B**) A dilution series of 22Rv1 input RNA to assay AR-V7 (FAM, blue) and total AR (HEX, green); (**A**) 1D ddPCR graph and (**B**) copy numbers; (**C**,**D**) a dilution series of 22Rv1 input RNA in 2000 pg lymphocyte WME-099 RNA to assay AR-V7 (FAM, blue) and AR (HEX, green); (**C**) 1D ddPCR graph and (**D**) copy numbers. Error bars represent 95% confidence intervals. In 1D dotblots, samples with the same amount of 22Rv1 input RNA are separated by black dotted lines, duplicates by yellow dotted lines; RNA concentration inputs within the range predicted for a single cell, ~10–30 pg, are highlighted in pink.

**Figure 3:**
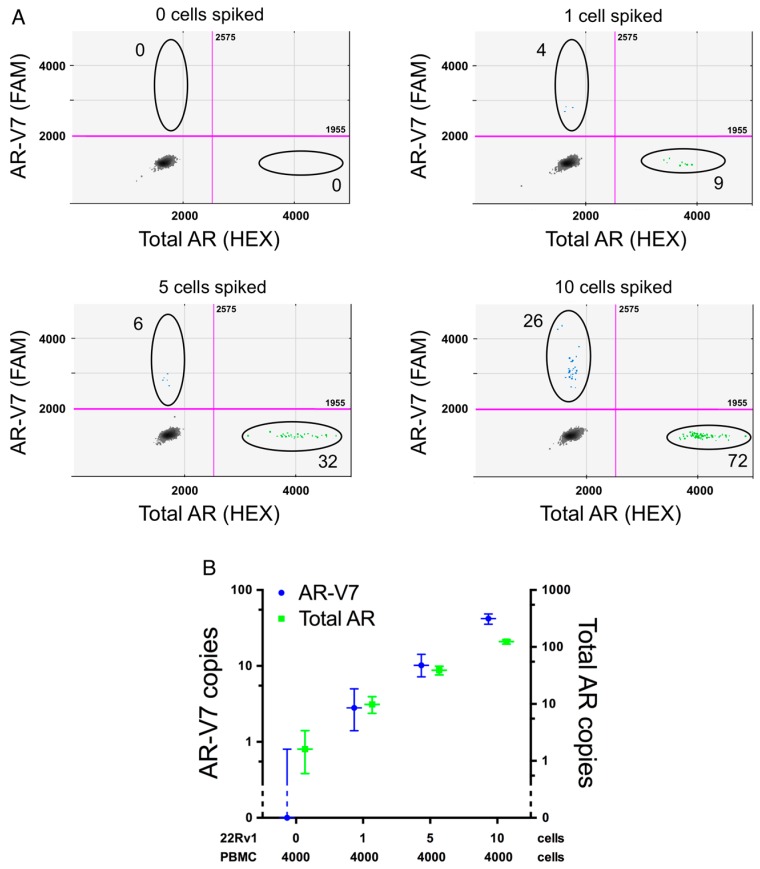
Modeled CTC samples. Prostate cancer CTC samples were modeled by spiking indicated numbers of 22Rv1 cells into 4000 lymphocytes from healthy donors by micro-manipulation using the CellCelector (ALS, Jena, Germany). Data was derived from three independent experiments. (**A**) Representative 2D ddPCR plots from spike-in experiment (0, 1, 5, 10 22Rv1 cells, black circles highlight AR-V7 and total AR events); (**B**) copy numbers of total AR and AR-V7 dependent on 22Rv1 cell number.

One mammalian cell contains approximately 10–30 pg of total RNA dependent on cell type and physiological state [26]. To test the sensitivity of our new ddPCR assay we titrated down the amount of purified total input RNA from AR-V7 positive 22Rv1 cells to approximately the amount expected from a single cell (2000 down to 15.5 pg) either alone, or by dilution in a constant amount of AR-V7-negative total RNA from WME-099 cells as would be expected in an RNA extract from a CTC sample with residual lymphocytes. As presented in Figure 2, the sensitivity of total AR and AR-V7 detection is able to capture the input RNA expected from a single cell regardless of lymphocyte RNA background. This was also validated in a similar dilution series with RNA template from VCaP cells despite the lower expression of AR-V7 in these cells (data not shown). To more thoroughly define what number of PCa CTCs is required in a typical IsoFlux-processed sample to reliably detect AR-V7 and total AR expression with our AR-V7 assay, we spiked defined numbers of 22Rv1 cells into 4000 PBMCs. This lymphocyte number is based on the average total cell count post IsoFlux CTC enrichment (see Table 3). The resulting samples were processed for ddPCR AR-V7 screening in the same way as IsoFlux enriched patient CTC samples, with Figure 3 demonstrating that our assay reliably detects relevant AR-V7 expression from one spiked cell into 4000 lymphocytes. However this is only true in two out of three replicates. Whether this reflects true technical assay limitations, such as ability to consistently reverse transcribe RNA and synthesize cDNA, or whether in fact not all 22Rv1 cells express AR/AR-V7, remains speculation at present. The latter is supported by our data which show that statistically some cell lines express less than one copy AR-V7 per cell (Table 1), as well as previous AR-V7 detection in only 74% of individual 22Rv1 cells analyzed by quantitative PCR (qPCR) [27]. Thus expression of AR and its variants may be regulated by physiological events, such as cell cycle dependent regulation; indeed AR has been proposed to be transcriptionally repressed by the retinoblastoma protein [28]. This may also help in explaining that although AR reportedly can be lost in vivo in advanced prostate cancer tissue, AR-loss was to our knowledge only found in a heterogeneous manner [29]. Consequently, a conservative interpretation of the assay in our hands is that we can confidently detect AR-V7 status in the presence of at least five prostate cancer CTCs. This allows, even in the presence of tumor cell heterogeneity, reproducible detection of enough ddPCR events positive for total AR, or importantly, AR-V7. Thus, our AR-V7 detection assay with ddPCR has high specificity and sensitivity, sufficient to reliably define AR-V7 expression for most of our advanced PCa patient samples, which have a median CTC number of 32 (range: 3–184, Table 3). By contrast, detection efficiency for other methods has not been documented in detail in most other studies but we note that AR-V7 was detected in samples with a range of one to a minimum of ten CTCs [18,27].

### 2.4. AR-V7 Expression in Patient Circulating Tumor Cell (CTC) Samples

The ddPCR assay was validated by testing twenty six CTC samples from twenty-four PCa patients after IsoFlux CTC isolation for expression of AR-V7. AR-V7 was detected in 30.8% of CTC samples (8/26): no AR-V7 was detected in any (0/10) of the HSPC (hormone sensitive prostate cancer) samples (note, HSPC patient 3 was with only three CTCs below our conservatively estimated AR-V7 detection limit) whereas AR-V7 was detected in 50% (8/16) of CRPC samples. Detection of AR-V7 in CTC positive patient samples is within the range reported by others for qPCR AR-V7 assays (29%–55%) and above that found by immunocytostaining (18%) [13,16,17,18,27]. As expected, residual lymphocyte counts had no effect on detectability of either form of the AR in our assay. AR-V7 expression in positive patient samples ranged from 8 to 1632 copies per eight mL blood. Patient 18 with eight AR-V7 copies had a CTC count of 47, highlighting tumor cell heterogeneity since clearly not all cells contribute to AR-V7 expression, a finding also observed for patient 19 who had sixteen AR-V7 copies detected from 70 CTCs. The lowest CTC count in a patient sample with detectable AR-V7 was ten (patient 20). Since we detected as many as 104 copies of AR-V7 from only 10 CTCs in this sample, it is likely our assay will detect AR-V7 in samples with considerably lower CTC counts as long as these cells express AR-V7. We will need to analyze larger patient cohorts to confirm this finding, but for now a minimum of five CTCs remains our conservatively estimated detection limit to confidently call a sample AR-V7-negative, based on our 22Rv1 spiking data and expected CTC heterogeneity. Interestingly, for two patients we tested samples taken approximately 3 month apart (patient 16 and 17). Both patients were considered to have CRPC at both time points, but patient 17 changed from AR-V7 negative to positive. He had a PSA of 76.6 at the first CTC sampling but showed disease progression radiologically, as well as resistance to Abiraterone with an increase of his PSA to 193.7 by the time his second sample was taken. Patient data are presented in Table 3. Despite the small patient cohort studied here for method validation, Table 4 summarizes that AR-V7 detection significantly correlated with CRPC (*p* = 0.008).

## 3. Materials and Methods

### 3.1. Cell Lines

The human PCa cell lines LNCaP, VCaP, C4-2, C4-2B, PC3, LAPC, 22Rv1, as well as the human b-lymphocyte cell line WME-099, were either recently purchased (ATCC, in vitro Technologies, Lane Cove, Australia) or authenticated (AGRF, Melbourne, Australia). Cells were maintained in RPMI media (Lonza, Basel, Switzerland) supplemented with 10% FBS (Invitrogen, Carlsbad, CA, USA) in a humidified incubator with 5% CO_2_ at 37 °C.

### 3.2. Patients

Twenty-four patients diagnosed with high risk PCa and positive for CTCs were recruited at Liverpool Hospital. Of the twenty-four patients, twenty-two had metastatic disease, with the remaining two having biochemical recurrent disease (Gleason grade 7 or greater). All patients and healthy blood donors gave informed consent to participate in the study. The study was conducted in accordance with the Declaration of Helsinki, and the protocol was approved by the South Western Sydney Local Health District Ethics Committee (Ref: HREC/13/LPOOL/158, 2nd September 2013). Whole blood from patients or healthy donors was drawn in 9 mL K3E K3EDTA-tubes (Greiner Bio-one, Kremsmünster, Austria) after discarding the first 2 mL blood to avoid contamination with epithelial skin cells. Patients were considered to have CRPC if they had experienced new metastases, progression of metastases or a rise in PSA despite adequate castration serum testosterone levels <1.7 nmol/L [30].

### 3.3. CTC Enrichment

CTC enrichment was performed with the IsoFlux CTC enrichment kit (Fluxion, San Francisco, CA, USA) according to manufacturer’s instructions. Briefly, PBMCs were separated from 8 mL of blood using 50 mL SepMate tubes and Lymphoprep (STEMCELL, Melbourne, Australia) according to the manufacturer’s instructions. PBMCs were once washed with PBS and transferred into a 1.5 mL Low-Protein-Bind tubes (Eppendorf, Hamburg, Germany) with 40 µL anti-human EpCAM conjugated immunomagnetic beads and 40 µL Fc blocker and incubated at 4 °C with slow agitation for 1.5 h. Cells were then loaded into isolation cartridges and CTCs were isolated using the IsoFlux CTC enrichment system and run using the standard separation protocol (Fluxion). Isolated CTCs were either enumerated or frozen at −80 °C for later down-stream analysis.

### 3.4. Immunocytostaining and CTC Enumeration

CTCs were immunocytostained for the presence of cytokeratin, CD45 and nuclei by Hoechst dye using the IsoFlux CTC enumeration kit (Fluxion) according to the manufacturer’s instructions. CTCs mounted in 24-well glass bottom plates were visualized and scanned with Olympus Ix71 (Olympus, Tokyo, Japan) mounted with an automated stage ProScan III (PRIOR) (10× objective). The exposure time for Hoechst, CD45 and cytokeratin are 2, 200 and 400 ms, respectively. CD45 negative cells with positive Hoechst and cytokeratin staining were considered to be CTCs and counted manually from scanned images. Total cell numbers (i.e., including residual blood cells) were also determined in the Hoechst channel using the Olympus CellSens Software “count and measure” plugin (Tokyo, Japan).

### 3.5. RNA Extraction and cDNA Synthesis

Total RNA from cell lines was extracted with the ISOLATE II RNA Mini Kit (Bioline, Sydney, Australia) and any residual genomic DNA contamination was removed by on-column DNAse I treatment for 15 min. RNA was eluted in 50 µL RNase-free H_2_O. RNA quality and quantity were measured using the NanoDrop 2000 (Thermo Scientific, Waltham, MA, USA). cDNA synthesis was performed from 1 µg total RNA with the SensiFAST cDNA Synthesis kit (Bioline, Sydney, Australia). Total RNA from IsoFlux CTC samples or healthy control PBMCs was extracted with the RNA purification Micro kit (Norgen Biotek Corp., Thorold, ON, Canada) and double-eluted in a total volume of 30 µL RNase-free H_2_O. 15 µL of this RNA was converted into cDNA with the SensiFAST cDNA Synthesis kit (Bioline). Healthy donor PBMCs consisted of 4000 cells to mimic IsoFlux CTC samples.

### 3.6. Droplet Digital PCR (ddPCR)

Primers and Taqman probes were designed using NCI primer software (Table 5). ddPCR samples for total AR and AR-V7 were set up with 20 µL reaction mixture containing 10 µL ddPCR Supermix for Probes, no dUTP (Bio-Rad, Hercules, CA, USA), 500 nM of each forward primer (FP) and reverse primer (RP) and 250 nM probe (FAM and HEX). Droplets were generated with 70 µL oil using a QX200 droplet generator (Bio-Rad). Amplification was performed at 95 °C, 10 min; followed by 40 cycles of 94 °C, 30 s and 55 °C (or 60 °C for actin) 1 min using a C1000 Touch thermocycler (Bio-Rad). After amplification, the droplets were read on a QX200 droplet reader (Bio-Rad) and analyzed with QuantaSoft software V1.7.4 (Bio-Rad). The total error calculated by the software was used as the 95% confidence intervals.

### 3.7. Modeling CTC Samples and Single Cell Micromanipulation

Single cells were isolated using the CellCelector (ALS GmbH, Jena, Germany) as described before [31]. In brief, 30 µm capillaries were used to pick live 22Rv1 cells under Brightfield at 20× magnification from 2% BSA-coated glass slides. Selected cells were aspirated with a volume of 20–100 nL and deposited into PCR tubes containing 100 µL RL buffer (ISOLATE II RNA Mini Kit, Bioline) and combined with 250 µL RL buffer containing 4000 healthy donor PBMCs. Samples were processed for RNA extraction and cDNA synthesis, as outlined for IsoFlux CTC samples.

### 3.8. Statistics

To establish the relationship between hormone sensitivity and AR-V7 detection, we performed a Fisher’s Exact Test using SPSS Statistics (IBM) software package version 23 (New York, NY, USA).

## 4. Conclusions

We developed a specific and sensitive ddPCR-based assay to determine AR-V7 expression in PCa CTC samples that can reliably detect AR-V7 expression from CTC positive patient samples. The advantage of the ddPCR based assay is that it determines not only AR-V7 positivity but actual transcript copy numbers and that allows in some instances to detect heterogeneity of AR-V7 gene expression without further separation of individual CTCs from residual lymphocytes and other CTCs after immunomagnetic CTC isolation using the IsoFlux platform. Although we were able to detect AR-V7 from a single cell, we conservatively estimate that AR-V7 can be reliably determined in samples with at least five CTCs, as this takes into account heterogeneity of AR-V7 expression as evident in two of our patient samples (patient 18 and 19). In our small proof of concept patient set, association of AR-V7 detection was significantly associated with development of CRPC and we are currently screening larger patient cohorts to confirm the robustness of our approach and association with clinical parameters. Our assay is likely adaptable for CTCs isolated by other methods or for circulating tumor RNA, which can be isolated from processed plasma samples.

## Figures and Tables

**Table 1:** Expression levels and ratio of AR-V7 and total AR in cell lines.

	22Rv1	VCaP	C4-2	LNCaP	C4-2B	LAPC4	PC3	WME099
**AR-V7 (copies/cell)**	4.6	1.2	0.5	0.4	0.4	0.0	0.0	0.0
**AR-V7** **95% CI**	2.6–6.6	0.8–1.8	0.2–0.9	0.1–0.8	0.1–0.7	0.0–0.2	0.0–0.3	0.0–0.2
**Total-AR (copies/cell)**	17.6	97.0	24.7	44.4	47.8	13.4	0.0	0.1
**Total-AR** **95% CI**	14.4–21.0	82.7–111.4	22.3–27.0	38.9–49.9	44.4–51.1	11.5–15.2	0.0–0.3	0.0–0.4
**V7/AR (%)**	26.0	1.3	1.9	0.9	0.7	0.0	0.0	0.0

Copies/cell were calculated from copies/µL droplet digital PCR (ddPCR) reaction data by accounting for the cDNA input corresponding to 500 pg RNA, and assuming 30 pg RNA/cell; CI: confidence interval.

**Table 2:** Negligible expression levels of AR-V7 and total AR in blood cells.

	PBMC-1	PBMC-2	PBMC-3	PBMC-4	PBMC-5	PBMC-6
**AR-V7 (copies/cell)**	0	0	0	0	0	0
**AR-V7 95% CI**	0–0.004	0–0.003	0–0.003	0–0.003	0–0.003	0–0.002
**Total-AR (copies/cell)**	0	0	0.002	0	0.001	0
**Total-AR 95% CI**	0–0.004	0–0.003	0–0.00	0–0.003	0–0.006	0–0.003

Four thousand peripheral blood mononuclear cells (PBMCs) per healthy donor were processed the same way as patient circulating tumor cell (CTC) samples to determine background AR-V7 and total AR expression. Copies/cell was calculated from copies/µL ddPCR reaction data by accounting for the cDNA input; CI: confidence interval.

**Table 3:** Patient data.

Hormone Sensitivity	Patient	AR-V7 Copies	Total AR Copies	%AR-V7 of Total AR	CTC Count	Total Cell Number
HSPC	1	0	96	0	31	2700
	2	0	0	n/a	7	2897
	3 *	0	0	n/a	3	6919
	4	0	0	n/a	7	6800
	5	0	40	0	9	3848
	6	0	0	n/a	56	3400
	7	0	80	0	8	3380
	8	0	0	n/a	7	2732
	9	0	8	0	6	5464
	10	0	24	0	65	7366
CRPC	11	0	296	0	25	3182
	12	0	360	0	28	6229
	13	0	0	n/a	102	3997
	14	0	88	0	35	1566
	15	0	16	0	184	3224
	16a	0	24	0	82	3715
	16b	0	960	0	81	2058
	17a	0	0	n/a	122	1163
	17b	32	1152	2.5	12	3686
	18	8	1000	0.8	47	1505
	19	16	768	2.3	70	1820
	20	104	5336	1.9	10	8900
	21	264	37,008	0.7	39	1418
	22	360	20,880	1.7	44	4600
	23	880	153,120	0.6	12	2077
	24	1632	74,824	2.2	56	4434

AR-V7 and total AR are normalized from template input to represent copy number per 8 mL blood sample. Patient 16 and 17 had consecutive samples evaluated (b) was analyzed ~3 months following sample (a). n/a: not applicable; * note, patient 3 has only 3 detected CTCs and is, thus, below our conservatively estimated AR-V7 detection limit.

**Table 4:** AR-V7 status of circulating tumor cells (CTCs) correlates to hormone resistance.

AR-V7	HSPC	CRPC	Total
+ve	0	8	8
−ve	10	8	18
total	10	16	26

Association of AR-V7 with CRPC is statistically significant *p* = 0.008. Two of fourteen CRPC patients were analyzed at two time points (three month intervals) with one of them changing from AR-V7 negative to positive for the second time point. (total = total sample number; +ve: positive; −ve: negative).

**Table 5:** Primers and probes.

AR-Species	Primers	Probes
**Total AR**	FP: 5′-GGAATTCCTGTGCATGAAAGC-3′	5′-[HEX]CTTCAGCATTATTCCAGTG[BHQ1]-3′
	RP: 5′-CGATCGAGTTCCTTGATGTAGTTC-3′	
**AR-V7**	FP: 5′-CGGAAATGTTATGAAGCAGGGATGA-3′	5′-[6FAM]CGGAATTTTTCTCCCAGA[BHQ1]-3′
	RP: 5′-CTGGTCATTTTGAGATGCTTGCAAT-3′	

## Abbreviations

AR	androgen receptor
PCa	prostate cancer
CRPC	castrate resistant prostate cancer
HSPC	hormone sensitive prostate cancer
CTC	circulating tumor cell
ctNA	circulating tumor nucleic acid
PSA	prostate specific antigen
CEA	carcinoembryonic antigen
EGFR	epidermal growth factor receptor
ADT	androgen deprived therapy
EpCam	epithelial cell adhesion molecule
CK	cytokeratin
PBMC	peripheral blood mononuclear cell
ddPCR	droplet digital PCR
FP	forward primer
RP	reverse primer
FISH	fluorescent in situ hybridysation
